# Supplementary material for: VWCE modulates amino acid-dependent mTOR signaling and coordinates with KICSTOR to recruit GATOR1 to the lysosomes
Source: Nat Commun. 2023 Dec 20;14:8464. doi: 10.1038/s41467-023-44241-8 (PMC10733324; doi:10.1038/s41467-023-44241-8)
Supplement: Supplementary file 1 — Supplementary Information [file 41467_2023_44241_MOESM1_ESM.pdf]

Supplementary Information

VWCE modulates amino acid-dependent mTOR signaling and coordinates with KICSTOR to recruit GATOR1 to the lysosomes

Zhao T. et al.

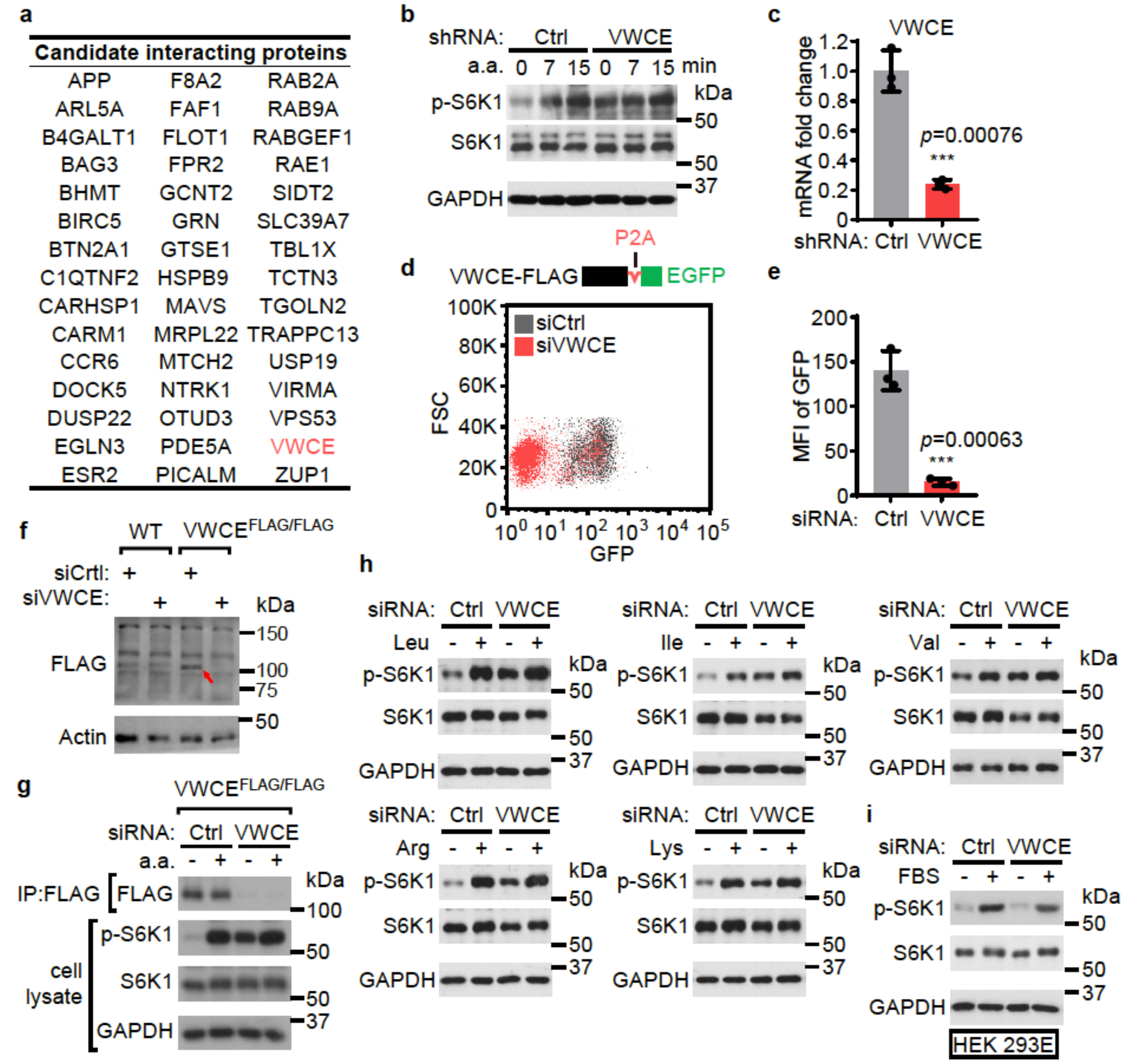

Supplementary Figure 1. VWCE specifically regulates amino acid-dependent mTORC1 activity. a, Candidate proteins for subsequent screening of regulators of the mTORC1 pathway. Based on the BioPlex and BioGRID database, each protein interacts with at least two components in the nutrient-sensing pathway that regulates mTORC1. **b**, Knockdown of VWCE by shRNA impairs the suppression of mTORC1 upon amino acid deprivation. HEK293T cells were deprived of amino acids for 60 min, or deprived and restimulated with amino acids for 7 or 15 min. **c**, The knockdown efficiency of VWCE in (b) was measured by RT-qPCR (N=3). Data are mean ± s.d., ***p < 0.001 (unpaired two-sided Student's t-test). **d**, The knockdown efficiency of VWCE in HEK293T cells was measured by flow cytometry. Cells endogenously expressing VWCE-FLAG-P2A-EGFP were transfected with siCtrl or siVWCE. **e**, The mean fluorescence intensity (MFI) of GFP in (d) are quantified across three independent replicates. Data are mean ± s.d., ***p < 0.001 (unpaired two-sided Student's t-test). **f**, Wild-type HEK293T cells or those with endogenously FLAG-tagged VWCE (VWCE$^{FLAG/FLAG}$) were transfected with siCtrl or siVWCE. The cell lysates were then enriched using 50 kD centrifugal filters and subjected to immunoblot analysis. The VWCE-FLAG protein band is marked by a red arrow. **g**, The VWCE$^{FLAG/FLAG}$ cells were transfected with siCtrl or siVWCE. The immunoprecipitates and cell lysates were then analyzed via immunoblotting. **h**, HEK293T cells transfected with siCtrl or siVWCE were starved

of a specific type of amino acid for 50 min (-), or starved and restimulated with the same amino acid for 10 min (+). **i**, HEK293E cells transfected with siCtrl or siVWCE were starved of FBS for 60 min (-), or starved and restimulated with 10% FBS for 20 min (+). The immunoblotting assays were independently replicated three times with consistent results. Source data are provided as Source Data files.

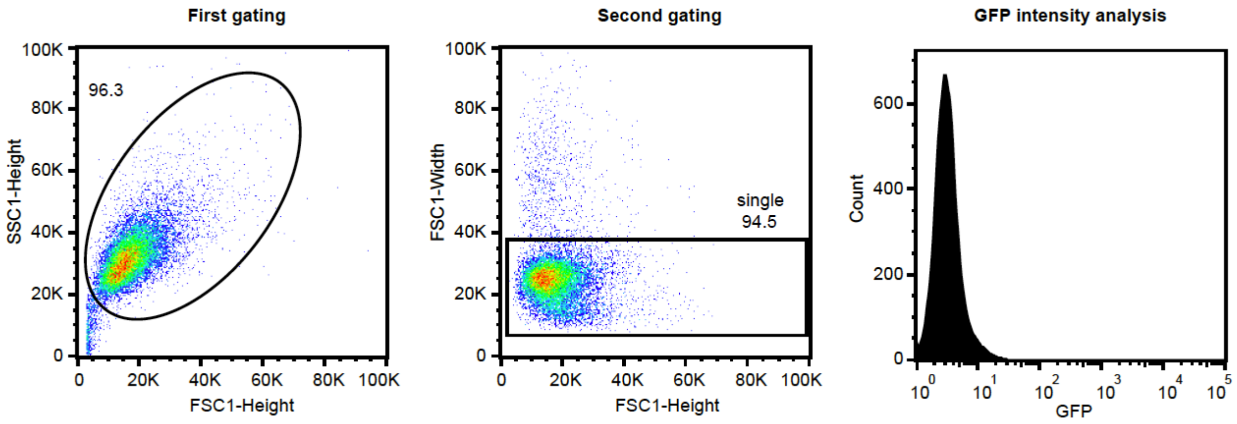

Supplementary Figure 2. Gating strategy of Flow Cytometry assay.

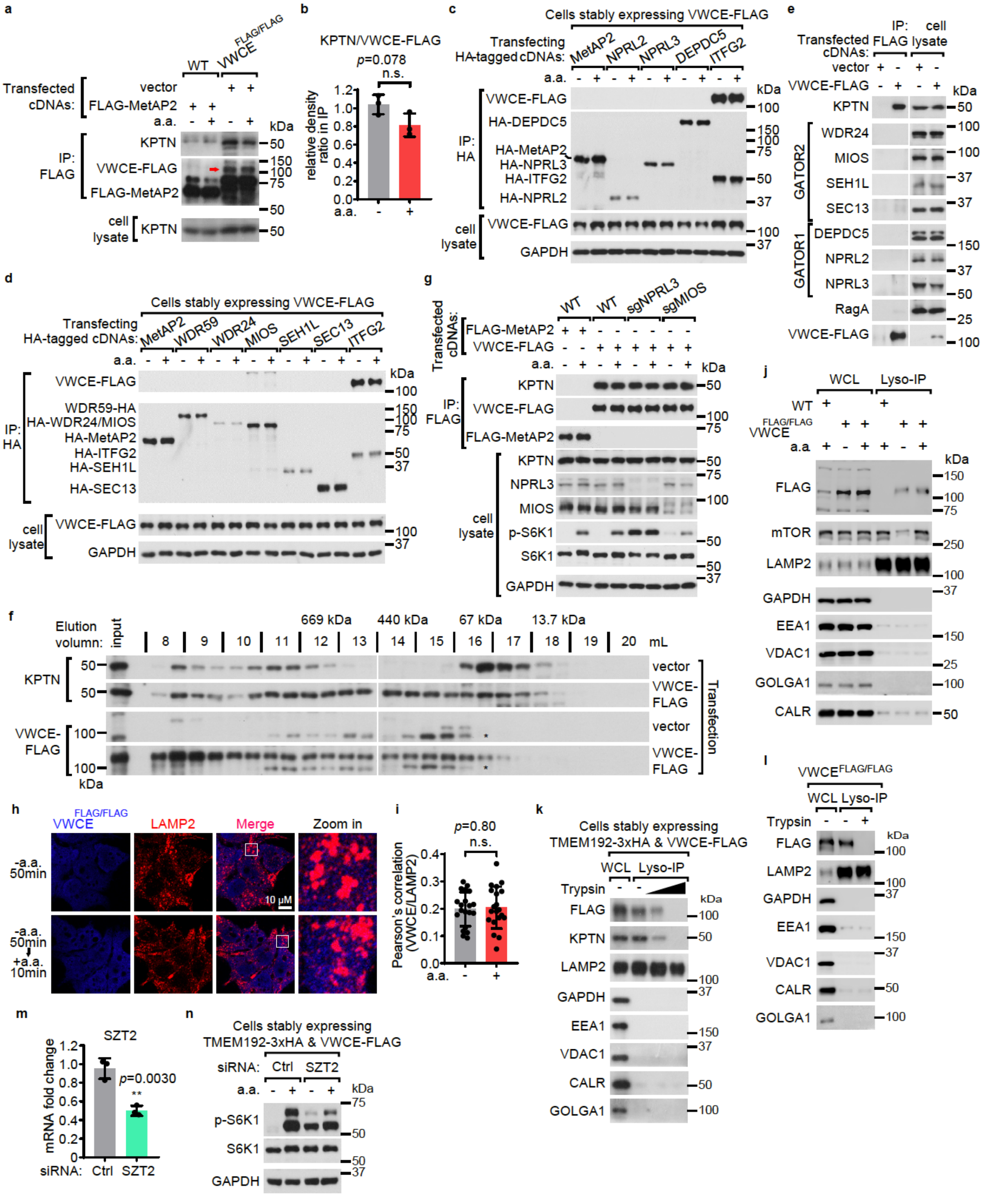

Supplementary Figure 3. VWCE interacts with the KICSTOR complex. **a**, Endogenous VWCE interacts with KPTN. Cells with endogenous FLAG-tagged VWCE (VWCE$^{FLAG/FLAG}$) were transfected with the indicated cDNAs. Bands of VWCE-FLAG are indicated by a red arrow. **b**, The relative levels of KPTN/VWCE-FLAG in the immunoprecipitates in (a) are quantified across three independent replicates. Data are mean ± s.d., n.s., not significant (unpaired two-sided Student's *t*-test). **c**, **d**, VWCE does not interact with GATOR1 (c) or GATOR2 (d). Cells stably expressing VWCE-FLAG were transfected with the indicated cDNAs. **e**, VWCE co-immunoprecipitates with the KICSTOR component KPTN, but not GATOR1 or GATOR2. **f**, Size-exclusion chromatography analysis was performed on KPTN and VWCE. Lysates were obtained

from HEK293T cells transfected with the indicated cDNAs, and subjected to a Superose 6 size-exclusion chromatography column. The asterisks denote nonspecific bands. **g**, VWCE-KPTN interaction is independent of GATOR1 or GATOR2. Wild-type (WT), NPRL3 or MIOS knockout cells were transfected with the indicated cDNAs. **h**, The amino acid-insensitive localization of VWCE on lysosomes was revealed by immunostaining of $VWCE^{FLAG/FLAG}$ cells. **i**, Quantification of the co-localization between VWCE and LAMP2 in (h) (N=20). Data are mean ± s.d. n.s., not significant (unpaired two-sided Student's *t*-test). **j**, Amino acid-insensitive localization of VWCE on lysosomes. Lysosomes were immunopurified from WT or $VWCE^{FLAG/FLAG}$ HEK293T cells that stably expressing 3×HA-tagged TMEM192. **k**, **l**, VWCE resides on the cytoplasmic face of lysosomes. Lysosomes immunopurified from HEK293T cells stably expressing VWCE-FLAG (k), or $VWCE^{FLAG/FLAG}$ cells (l) were either digested with trypsin [2.5 or 10 μg in (k); 10 μg in (l)] or left untreated. To detect endogenous VWCE protein in (l), both Lyso-IP and WCL samples were concentrated using 50 kD centrifugal filters. **m**, The knockdown efficiency of SZT2 was measured by RT-qPCR (N=3). Data are mean ± s.d., $^{**}p < 0.01$ (unpaired two-sided Student's *t*-test). **n**, Knockdown of SZT2 impairs the suppression of mTORC1 activity upon amino acid deprivation. The immunoblotting assays were independently replicated three times with consistent results. Source data are provided as Source Data files.

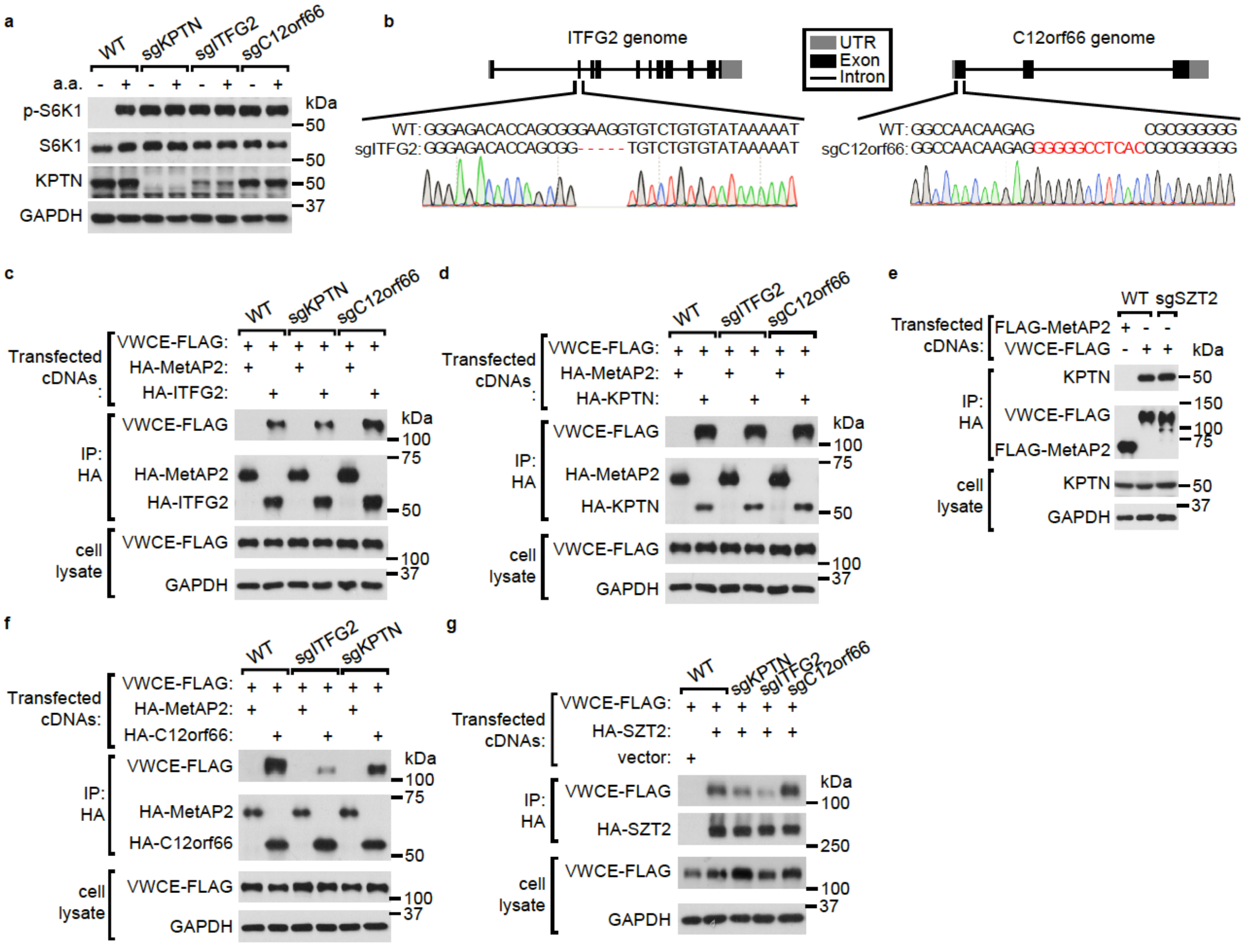

Supplementary Figure 4. VWCE interacts with KICSTOR through the KPTN/ITFG2 heterodimer. a, Validation of HEK293T cell clones deficient in indicated KICSTOR components. Knockout of KPTN, ITFG2, or C12orf66 impairs the suppression of mTORC1 activity upon amino acid deprivation. Cell lysates were analyzed by immunoblotting for the indicated proteins. **b**, Cells deficient in ITFG2 or C12orf66 were verified by DNA sequencing. The five-base deletion in ITFG2 or eleven-base insertion in C12orf66 causes frameshift mutations, resulting in prematurely terminated translation. **c**, The VWCE-ITFG2 interaction does not depend on KPTN or C12orf66. **d**, **e**, The VWCE-KPTN interaction does not depend on ITFG2, C12orf 66 (d) or SZT2 (e). **f**, Knockout of ITFG2 or KPTN impairs VWCE-C12orf66 interaction. **g**, The VWCE-SZT2 interaction is impaired by deficiency of KPTN or ITFG2, but not C12orf66. For c-g, both WT and KICSTOR component-deficient cells were transfected with the indicated cDNAs. The immunoprecipitates and cell lysates were then analyzed by immunoblotting. The immunoblotting assays were independently replicated three times with consistent results. Source data are provided as a Source Data file.

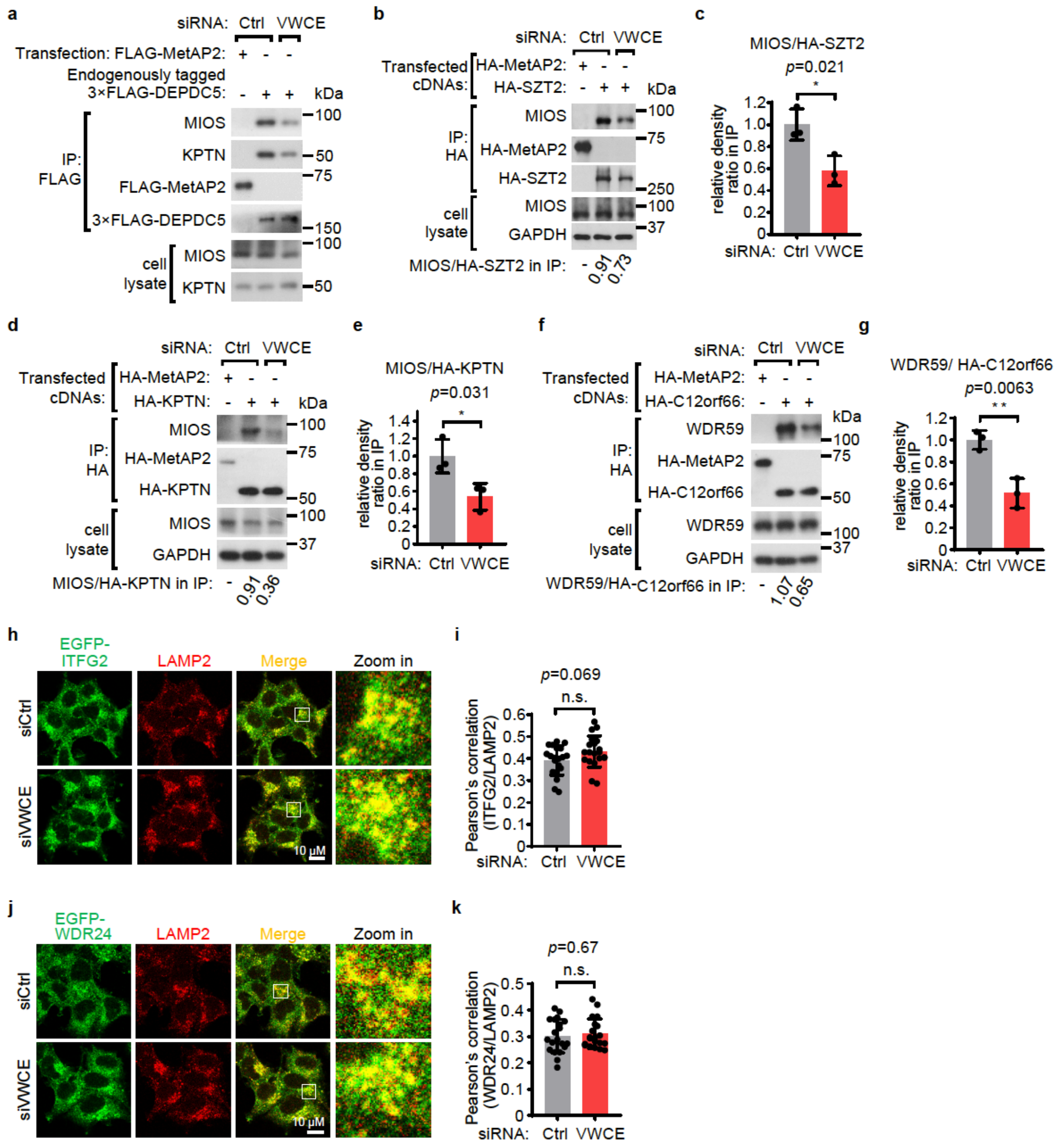

Supplementary Figure 5. Knockdown of VWCE impairs KICSTOR-GATORs interaction. a-g, Knockdown of VWCE impairs KICSTOR-GATOR2 interaction. Cells were transfected with the indicated cDNAs and siRNAs, then subjected to amino acid starvation (-) or starvation followed by restimulation (+). Cell lysates and immunoprecipitates (IPs) were analyzed by immunoblotting (a, b, d, f). The density ratios of the IPs in b, d, and f are quantified in c, e, and g, respectively (N=3). Data are mean ± s.d., **p* < 0.05; ***p* < 0.01 (unpaired two-sided Student's *t*-test). **h**, **j**, Knockdown of VWCE does not affect the lysosomal localization of KICSTOR or GATOR2. Cells stably expressing EGFP-ITFG2 or EGFP-WDR24 were immunostained with an anti-LAMP2 antibody to measure the co-localization of lysosomes and KICSTOR (h) or GATOR2 (j). **i**, **k**, Quantification of the co-localization in (h) and (j), respectively (N=20). Data are mean ± s.d. n.s., not significant (unpaired two-sided Student's *t*-test). The immunoblotting assays were independently replicated three times with consistent results. Source data are provided as Source Data files.

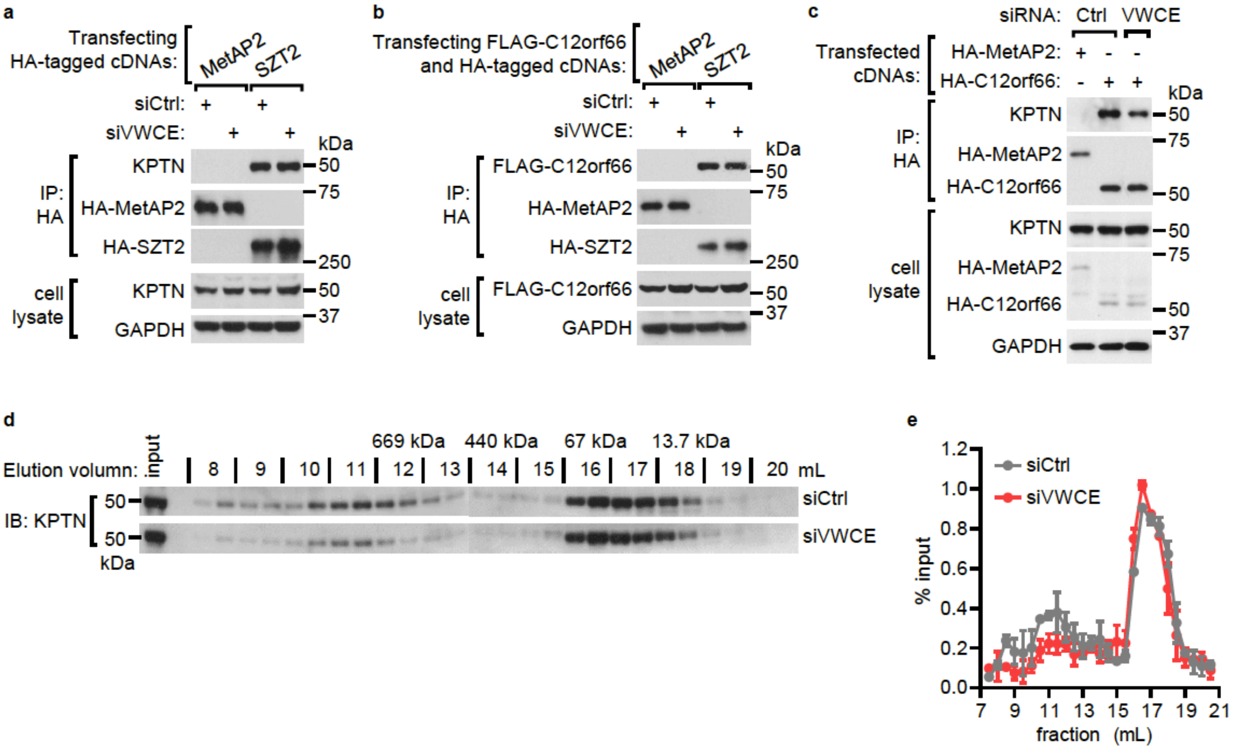

Supplementary Figure 6. Knockdown of VWCE impairs KICSTOR conformation. a, **b**, The interaction between SZT2 and KPTN (a) or C12orf66 (b) remains unaffected by VWCE knockdown. **c**, The KPTN-C12orf66 interaction is impaired by VWCE knockdown. For a-c, the immunoprecipitates and cell lysates from cells transfected with the indicated siRNAs or cDNAs were analyzed by immunoblotting. **d**, Size-exclusion chromatography was performed on the cell lysates, which were then analyzed by immunoblotting. HEK293T cells transfected with either siCtrl or siVWCE were lysed and the resulting solutions were fractionated using Superose 6 Increase 10/300 GL size-exclusion chromatography column. **e**, The density ratio is quantified as the signal of each fraction versus the input (N=2). Data are mean ± s.e.m. The immunoblotting assays were independently replicated three times with consistent results. Source data are provided as a Source Data file.

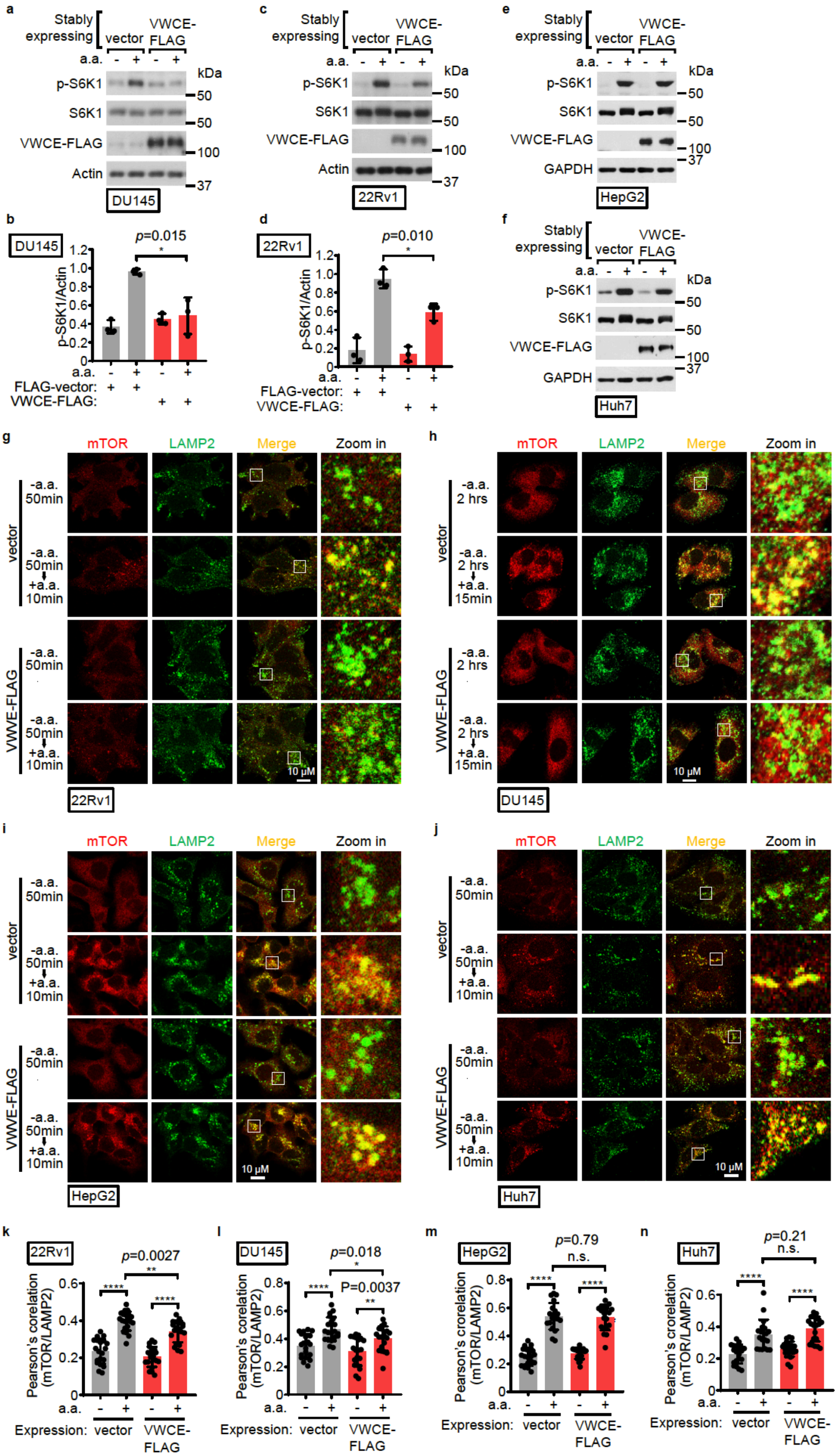
a
Stably expressing
vector
VWCE-FLAG
a.a.
p-S6K1
S6K1
VWCE-FLAG
Actin
kDa
50
50
100
37
DU145
b
DU145
p=0.015
p-S6K1/Actin
a.a.
FLAG-vector:
VWCE-FLAG:
c
22Rv1
p=0.010
d
e
HepG2
GAPDH
f
Huh7
g
mTOR
LAMP2
Merge
Zoom in
vector
VWVE-FLAG
-a.a. 50min
-a.a. 50min +a.a. 10min
10 μM
h
-a.a. 2 hrs
-a.a. 2 hrs +a.a. 15min
i
j
k
p=0.0027
Pearson's corelation (mTOR/LAMP2)
Expression:
l
p=0.018
P=0.0037
m
p=0.79
n.s.
n
p=0.21
n.s.

Supplementary Figure 7. VWCE inhibits mTORC1 activation in prostate cancers . a, **c**, **e**, **f**, Overexpression of VWCE inhibits amino acid-dependent mTORC1 activation in the prostate cancer cell lines DU145 (a) and 22Rv1 (c), but not in liver cancer cell lines HepG2 (e) and Huh7 (f). Cells stably expressing the indicated cDNAs were deprived of amino acids for 50 min (22Rv1, HepG2 and Huh7) or 2 hrs (DU145) (-), or deprived and restimulated with amino acids for 10 min (22Rv1, HepG2 and Huh7) or 15 min (DU145) (+), before being lysed for immunoblotting. **b**, **d**, The relative levels of p-S6K1/Actin in (a, c) are quantified across three independent replicates. Data are mean ± s.d. *p <0.05 (unpaired two-sided Student's *t*-test). **g-j**, Stable expression of VWCE inhibits the lysosomal localization of mTOR in the prostate cancers (g, h), but not in liver cancers (i, j). The cells stably expressing either the vector or VWCE were treated as indicated and immunostained with anti-mTOR (red) and anti-LAMP2 (green) antibodies. **k-n**, Quantification of co-localization between mTOR and LAMP2 in (g-j) (N=20). Data are mean ± s.d. *P<0.05; **p <0.01; ****p <0.0001; n.s., not significant (unpaired two-sided Student's *t*-test). The immunoblotting assays were independently replicated three times with consistent results. Source data are provided as Source Data files.

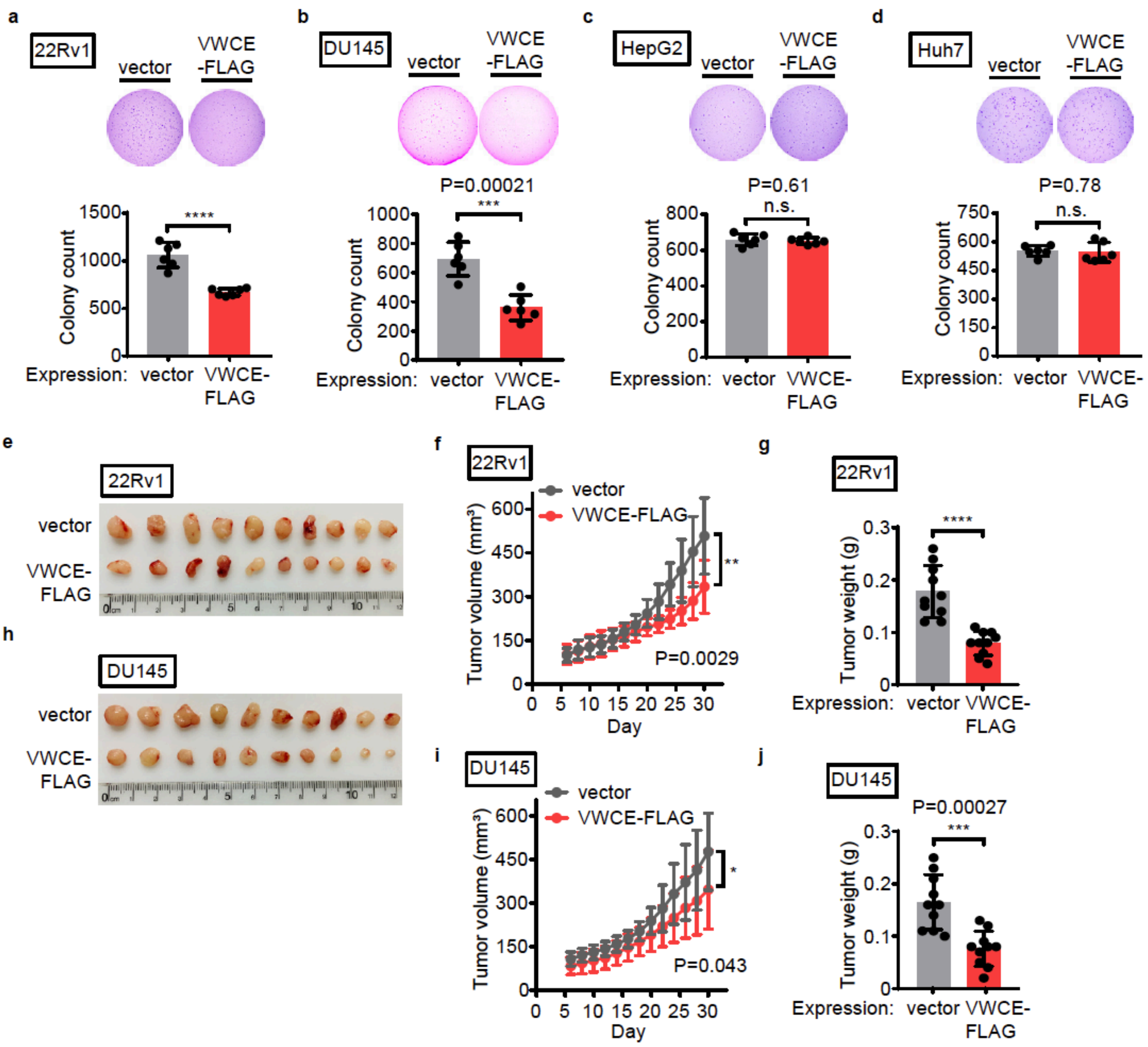

Supplementary Figure 8. Overexpression of VWCE inhibits prostate cancer development. a-d, Stable overexpression of VWCE inhibits anchorage-independent cell growth in prostate cancer cells (a, b), but not in liver cancer cells (c, d). A total of 5,000 22Rv1/ HepG2/ Huh7 cells or 10,000 DU145 cells were seeded into each well of a 6-well cell culture plate. The number of colonies were quantified (N=6). Data are mean ± s.d. ***p <0.001; ****p <0.0001; n.s., not significant (unpaired two-sided Student's *t*-test). **e-j**, Overexpression of VWCE inhibits subcutaneous xenograft tumor growth of 22Rv1 and DU145 cells. The cells stably expressing either the vector or VWCE were subcutaneously injected into nude mice for xenograft growth. Tumor images (e, h), tumor volumes (f, i), and tumor weights (g, j) are shown. Tumor volumes and tumor weights are quantified (N=10). Data are mean ± s.d. *p <0.05; **p <0.01; ***p < 0.001; ****p < 0.0001 (unpaired two-sided Student's t-test). Source data are provided as a Source Data file.

Supplementary Table 1: Candidate proteins identified through interaction analysis of the mTORC1 pathway components

Candidates for RNAi screening	Components in the mTORC1 signaling				
APP	KPTN	RagB			
ARL5A	NPRL2	NPRL3			
B4GALT1	DEPDC5	NPRL2	NPRL3		
BAG3	SEH1L	LAMTOR1			
BHMT	LAMTOR3	LAMTOR5			
BIRC5	LAMTOR5	LAMTOR4			
BTN2A1	KPTN	ITFG2			
C1QTNF2	SZT2	ITFG2			
CARHSP1	KPTN	ITFG2			
CARM1	DEPDC5	NPRL2			
CCR6	RagB	RagC			
DOCK5	TBL1X	SZT2			
DUSP22	LAMTOR4	LAMTOR1			
EGLN3	MIOS	WDR59	SEH1L		
ESR2	MIOS	LAMTOR2	LAMTOR4	LAMTOR1	SEC13
F8A2	KPTN	ITFG2			
FAF1	WDR24	WDR59	SEH1L	SEC13	MIOS
FLOT1	LAMTOR2	LAMTOR1			
FPR2	SLC38A9	RagB	RagC		
GCNT2	DEPDC5	NPRL3			
GRN	SAMTOR	RagA			
GTSE1	SEC13	KPTN			
HSPB9	KPTN	ITFG2			
MAVS	KPTN	LAMTOR1			
MRPL22	SEH1L	LAMTOR3			
MTCH2	LAMTOR1	NPRL2			
NTRK1	MIOS	WDR59			
OTUD3	KPTN	ITFG2			
PDE5A	MIOS	WDR24			
PICALM	SEC13	LAMTOR3			
RAB2A	LAMTOR3	RagC			
RAB9A	MIOS	RagA	LAMTOR3	LAMTOR1	LAMTOR5
RABGEF1	DEPDC5	NPRL2	NPRL3		
RAE1	MIOS	WDR24	WDR59	SEH1L	
SIDT2	LAMTOR1	LAMTOR4			
SLC39A7	DEPDC5	NPRL2	NPRL3		
TBL1X	TBL1X	NPRL2			
TCTN3	SLC38A9	ATP6V0C			
TGOLN2	RagB	LAMTOR1	SEC13		
TRAPPC13	DEPDC5	NPRL2	NPRL3		
USP19	LAMTOR4	KPTN			
VIRMA	SZT2	DEPDC5			
VPS53	DEPDC5	NPRL2	NPRL3		
VWCE	KPTN	ITFG2			
ZUP1	SZT2	C12orf66	DEPDC5		

Supplementary Table 2: Cancer type abbreviations

abbreviations	full names
BLCA	bladder urothelial carcinoma
BRCA	breast invasive carcinoma
CESC	cervical squamous cell carcinoma and endocervical adenocarcinoma
CHOL	cholangiocarcinoma
HNSC	head and neck squamous cell carcinoma
KICH	kidney chromophobe
PAAD	pancreatic adenocarcinoma
PCPG	pheochromocytoma and paraganglioma
PRAD	prostate adenocarcinoma
LIHC	liver hepatocellular carcinoma
