# Supplementary material for: VWCE modulates amino acid-dependent mTOR signaling and coordinates with KICSTOR to recruit GATOR1 to the lysosomes
Source: Nat Commun. 2023 Dec 20;14:8464. doi: 10.1038/s41467-023-44241-8 (PMC10733324; doi:10.1038/s41467-023-44241-8)
Supplement: Supplementary file 3 — Reporting Summary [file 41467_2023_44241_MOESM3_ESM.pdf]

nature portfolio

Corresponding author(s): Ying Liu

Last updated by author(s): Nov 20, 2023

Reporting Summary

Nature Portfolio wishes to improve the reproducibility of the work that we publish. This form provides structure for consistency and transparency in reporting. For further information on Nature Portfolio policies, see our Editorial Policies and the Editorial Policy Checklist.

Statistics

For all statistical analyses, confirm that the following items are present in the figure legend, table legend, main text, or Methods section.

n/a	Confirmed	
☐	☒	The exact sample size (*n*) for each experimental group/condition, given as a discrete number and unit of measurement
☐	☒	A statement on whether measurements were taken from distinct samples or whether the same sample was measured repeatedly
☐	☒	The statistical test(s) used AND whether they are one- or two-sided<br>*Only common tests should be described solely by name; describe more complex techniques in the Methods section.*
☒	☐	A description of all covariates tested
☐	☒	A description of any assumptions or corrections, such as tests of normality and adjustment for multiple comparisons
☐	☒	A full description of the statistical parameters including central tendency (e.g. means) or other basic estimates (e.g. regression coefficient) AND variation (e.g. standard deviation) or associated estimates of uncertainty (e.g. confidence intervals)
☐	☒	For null hypothesis testing, the test statistic (e.g. F, t, r) with confidence intervals, effect sizes, degrees of freedom and P value noted<br>*Give P values as exact values whenever suitable.*
☒	☐	For Bayesian analysis, information on the choice of priors and Markov chain Monte Carlo settings
☒	☐	For hierarchical and complex designs, identification of the appropriate level for tests and full reporting of outcomes
☐	☒	Estimates of effect sizes (e.g. Cohen's *d*, Pearson's *r*), indicating how they were calculated

Our web collection on statistics for biologists contains articles on many of the points above.

Software and code

Policy information about availability of computer code

Data collection	RT-qPCR data were collected using Bio-Rad CFX manage, version 3.1.1517.823.<br>Confocal images were collected using Zeiss LSM LSM980 with Airyscan2, 880 META UV/Vis or LSM 710 Zeiss confocal Microscope.<br>FACS data were collected using a Beckman Astrios EQ flow cytometer.<br>Size-exclusion chromatography was performed using an ATKA purifier (AKTA pure, GE Healthcare).
Data analysis	All statistical analyses were performed using GraphPad Prisim, version 7.04.<br>Quantification of confocal images and clone formation images were performed using Fiji software, version 1.0.<br>Flow cytometry analyses were performed using FlowJo V10.<br>Statistical analysis of TCGA data was performed using R (version 4.0.3) and R packages.<br>Quantifications of confocal images were performed using Fiji software (version 1.0) coupled with the Colocalization_Finder plugin.

For manuscripts utilizing custom algorithms or software that are central to the research but not yet described in published literature, software must be made available to editors and reviewers. We strongly encourage code deposition in a community repository (e.g. GitHub). See the Nature Portfolio guidelines for submitting code & software for further information.

Data

Policy information about availability of data

All manuscripts must include a data availability statement. This statement should provide the following information, where applicable:

- Accession codes, unique identifiers, or web links for publicly available datasets
- A description of any restrictions on data availability
- For clinical datasets or third party data, please ensure that the statement adheres to our policy

Source data are provided with this paper. The information about molecular features of VWCE was from UniProt database (https://www.uniprot.org/). The interaction information for siRNA screening in Supplementary Table 1 was from BioGRID (https://thebiogrid.org/) and BioPlex (https://bioplex.hms.harvard.edu/) databases. FPKM-normalized mRNA expression data (Fig. 4a) from The Cancer Genome Atlas (TCGA) was obtained from UCSC Xena data hub (https://xenabrowser.net/hub/). The mRNA expression data of cancer cell lines from CCEL project (Fig. 4b) was obtained from DepMap Portal (https://depmap.org/portal/download/all/). All other data supporting the findings of this study are available from the corresponding authors upon reasonable request.

Human research participants

Policy information about studies involving human research participants and Sex and Gender in Research.

Reporting on sex and gender	The study did not involve human research participants.
Population characteristics	See above.
Recruitment	See above.
Ethics oversight	See above.

Note that full information on the approval of the study protocol must also be provided in the manuscript.

Field-specific reporting

Please select the one below that is the best fit for your research. If you are not sure, read the appropriate sections before making your selection.

☒ Life sciences ☐ Behavioural & social sciences ☐ Ecological, evolutionary & environmental sciences

For a reference copy of the document with all sections, see nature.com/documents/nr-reporting-summary-flat.pdf

Life sciences study design

All studies must disclose on these points even when the disclosure is negative.

Sample size	No statistical methods were used to predetermine sample size. Sample sizes were chosen according to standard practices in the relevant field. Sample sizes for confocal microscropy were chosen from the example of previous publication (PMD: 35266843). The mouse sample size in our experiments were determined according to similar analysis in the literature (Toshiro Moroishi et al., 2016, Cell).
Data exclusions	For subcutaneous xenograft tumor growth assay, mice with evidently ulcerated tumors before the last day were euthanized and the corresponding tumor data were not included. This exclusion criteria has been established by IACUC at Peking University (FT-LiuY-5) where we conducted our assay.
Replication	All experiments were replicated at least twice and reached similar results.
Randomization	For all experiments, samples or mice were randomly allocated into groups
Blinding	Investigators were blinded to group allocation of mice during data collection and analysis. For other experiments, each experiment was repeated by different investigators and reached similar results.

Reporting for specific materials, systems and methods

We require information from authors about some types of materials, experimental systems and methods used in many studies. Here, indicate whether each material, system or method listed is relevant to your study. If you are not sure if a list item applies to your research, read the appropriate section before selecting a response.

Materials & experimental systems

n/a	Involved in the study
☐	☒ Antibodies
☐	☒ Eukaryotic cell lines
☒	☐ Palaeontology and archaeology
☐	☒ Animals and other organisms
☒	☐ Clinical data
☒	☐ Dual use research of concern

Methods

n/a	Involved in the study
☒	☐ ChIP-seq
☐	☒ Flow cytometry
☒	☐ MRI-based neuroimaging

Antibodies

Antibodies used

Antibodies against pS6K1 T389 (9234, 1:1500 for immunoblotting), S6K1 (9202, 1:1000 for immunoblotting), pAKT1 S473 (4060, 1:1000 for immunoblotting), mTOR (2983, 1:1000 for immunoblotting, 1:400 for immunostaining), RagA (4357, 1:1000 for immunoblotting), RagC (3360, 1:1000 for immunoblotting), NPRL2 (37344, 1:1000 for immunoblotting), WDR59 (53385, 1:1000 for immunoblotting), MIOS (13557, 1:500 for immunoblotting), CALR (12238, 1:1000 for immunoblotting), HA (3724, 1:2000 for immunoblotting, 1:50 for immunostaining), EEA1 (3288, 1:1000 for immunoblotting) were from Cell Signaling Technology; antibodies against SEC13 (sc-514308, 1:1000 for immunoblotting), AKT1 (sc-5298, 1:1000 for immunoblotting), LAMP2 (sc-18822, 1:1000 for immunoblotting, 1:300 for immunostaining), GOLGA1 (sc-59820, 1:1000 for immunoblotting), Prohibitin (sc-28259, 1:1000 for immunoblotting) were from Santa Cruz Biotechnology; antibodies against KPTN (16094-1-AP, 1:1000 for immunoblotting), WDR24 (20778-1-AP, 1:1000 for immunoblotting), VDAC1 (55259-1-AP, 1:1000 for immunoblotting) were from ProteinTech; antibodies against VWCE (ab184772, 1:300 for immunoblotting), DEPDC5 (ab213181, 1:500 for immunoblotting), SEH1L (ab218531, 1:1000 for immunoblotting), GAPDH (ab128915, 1:5000 for immunoblotting) were from Abcam; antibody against Actin (ACTB; AC026, 1:20000 for immunoblotting) was from Abclonal; antibodies against NPRL3 (HPA011741, 1:1000 for immunoblotting), FLAG (F7425, 1:3000 for immunoblotting, 1:50 for immunostaining; F1804, 1:1000 for immunoblotting), MYC (M4439, 1:2000 for immunoblotting) were from Sigma. The secondary antibody anti-rabbit HRP (7074, 1:20000 for immunoblotting) was from Cell Signaling Technology and the secondary antibody anti-mouse HRP (A4416, 1:10000 for immunoblotting) was from Sigma. Secondary antibodies anti-mouse Alexa Fluor 488 (green, A11029, 1:1000 for immunostaining) and 594 (red, A11032, 1:1000 for immunostaining), and anti-rabbit Alexa Fluor 594 (A11037, 1:1000 for immunostaining) and 405 (blue, A48254, 1:1000 for immunostaining) were from ThermoFisher.

Validation

All commercial antibodies were validated by the manufactures as indicated on their official websites which are accessible for the public.

Validation information summarized from Cell Signaling Technology (Species abbreviation: H-Human, M-Mouse, R-Rat, Mk-Monkey. Mi-Mink, Dm-D. melanogaster, Z-Zebrafish, B-Bovine, Sc-S. cerevisiae, All-All Species Expected. Application abbreviation: WB-Western Blot, IP-Immunoprecipitation, HC-Immunohistochemistry, ChlP-Chromatin immunoprecipitation, IF-Immunofluorescence. F-Flow Cytometry).
pS6K1 T389 (9234). Species reactivity: H M R Mk; Application: WB
S6K1 (9202). Species reactivity: H M R Mk; Application: WB, IP
pAKT1 S473 (4060): Species reactivity: H M R Hm Mk Dm Z B; Application: WB, IP, IHC, IF, F
mTOR (2983). Species reactivity: H M R Mk; Application: WB, IP, IHC, IF, F
RagA (4357). Species reactivity: H M R Mk; Application: WB, IP
RagC (3360). Species reactivity: H M R Mk; Application: WB, IP, IF
NPRL2 (37344). Species reactivity: H M R Mk; Application: WB, IP
WDR59 (53385). Species reactivity: H Mk; Application: WB, IP
MIOS (13557). Species reactivity: H M R; Application: WB, IP
CALR (12238). Species reactivity: H M R; Application: WB, IF, F
HA (3724). Species reactivity: All; Application: WB, IP, IHC, IF, F, ChIP
EEA1 (3288). Species reactivity: H M R; Application: WB, IP, IF

Validation information copied from Santa Cruz Biotechnology (Application abbreviation: WB-Western Blot, IP-Immunoprecipitation, IHC-Immunohistochemistry, IF-Immunofluorescence, FCM-Flow Cytometry).
LAMP2 (sc-18822), "recommended for detection of LAMP-2 of human origin by WB, IP, F, IHC (P) and FCM"
GOLGA1 (sc-59820), "recommended for detection of golgin 97 of broad species origin by WB, IP, IF, FCM and ELISA"
Sec13 (sc-514308), "recommended for detection of SEC13 of mouse, rat and human origin by WB, IP, IF and ELISA"
AKT1 (sc-5298),"recommended for detection of Akt1 of mouse, rat and human origin by WB, IP, IF, IHC(P), FCM and ELISA"
Prohibitin (28259), "Western blot analysis of Prohibitin in A-431 and Ramos are validated"

Validation information summarized from ProteinTech (Application abbreviation: WB-Western Blot, IP-Immunoprecipitation, IHC-Immunohistochemistry, IF-Immunofluorescence, FC-Flow Cytometry).
VDAC1 (55259-1-AP). Tested Reactivity: Human, Mouse, Rat; Tested Applications: WB, IP, IHC, IF
WDR24 (20778-1-AP). Tested Reactivity: Human, Mouse; Tested Applications: WB, IP, IHC, IF
KPTN (16094-1-AP). Tested Reactivity: Human, Mouse, Rat; Tested Applications: WB, IHC, IF

Validation information summarized from Abcam (Application abbreviation: WB- immunoblotting, IP-immunoprecipitation, lHC-Immunohistochemistry, IF-immunofluorescence, ICC-immunocytochemistry, EM-Electron Microscopy).
VWCE (ab184772). Application: ICC/IF, IHC-P. Reactivity: Human. The application of WB was verified by knockdown and overexpression assays by our lab.
DEPDC5 (ab213181). Application: WB; Reactivity: Mouse, Human
SEH1L (ab218531). Application: IP, WB; Reactivity: Mouse, Human
GAPDH (ab128915). Application: WB, IP, ICC/IF, IHC-P, Flow Cyt. Reactivity: Human, African green monkey

Validation information summarized from Abclonal (Application abbreviation: WB-immunoblotting, IHC-immunohistochemistry, IF-immunofluorescence, ICC-immunocytochemistry).
Actin (ACTB; AC026). Application: WB. Reactivity: Human, Mouse, Rat, Chicken, Zebrafish, Pig

Validation information summarized from Sigma (Application abbreviation: WB-immunoblotting, IP-immunoprecipitation, IHC-immunohistochemistry, IF-immunofluorescence, ICC-immunocytochemistry, ARR-microarray),
NPRL3 (HPA011741). Application: IF, IHC; Reactivity: human
MYC (M4439). Application: ARR, ICC, IH, IP, WB; Reactivity: human
Flag (F1804). Application: WB, IP, IHC, ICC, IF; Reactivity: all
FLAG (F7425). Application: DB, IF, IP, WB CL; Reactivity: all

Eukaryotic cell lines

Policy information about cell lines and Sex and Gender in Research

Cell line source(s)	HEK293T (#CRL-3216) and HEK293E (#CRL-10852) were from ATCC, HepG2 was from Prof. Lei Chen (Peking University), Huh7 was from Prof. Xiao-Wei Chen (Peking University), and prostate cancer cell lines (PC3, 22Rv1, and DU145) were from Prof. Hong Wu (Peking University).
Authentication	HEK293T and HEK293E were verified by ATCC via Short Tandem Repeat (STR). Other cells were not authenticated in our laboratory.
Mycoplasma contamination	No mycoplasma contamination was detected.
Commonly misidentified lines (See ICLAC register)	No commonly misidentified cell lines in ICLAC were used in this study.

Animals and other research organisms

Policy information about studies involving animals; ARRIVE guidelines recommended for reporting animal research, and Sex and Gender in Research

Laboratory animals	Six-week-old BALB/c nude mouse were brought from Charles River and kept in a specific pathogen-free facility. Mice were kept under ~20 ? with ~50% humidity in a 14-hour light/ 10-hour dark cycle.
Wild animals	The study did not involve wild animals.
Reporting on sex	Sex was not considered in the study design.
Field-collected samples	The study did not involve samples collected from field.
Ethics oversight	The animal study protocols were evaluated and approved by the Institutional Animal Care and Use Committee (IACUC, FT-LiuY-5) at Peking University.

Note that full information on the approval of the study protocol must also be provided in the manuscript.

Flow Cytometry

Plots

Confirm that:

☒ The axis labels state the marker and fluorochrome used (e.g. CD4-FITC).

☒ The axis scales are clearly visible. Include numbers along axes only for bottom left plot of group (a 'group' is an analysis of identical markers).

☒ All plots are contour plots with outliers or pseudocolor plots.

☒ A numerical value for number of cells or percentage (with statistics) is provided.

Methodology

Sample preparation	The HEK293T cells expressing endogenous VWCE-FLAG-P2A-EGFP were transfected with siCtrl or siVWCE. After 72-hr transfection, the cells were trypsinized and suspended in PBS supplemented with 2% FBS.
Instrument	BeckMan Astrios EQ flow cytometer
Software	FlowJo V10
Cell population abundance	Cells was analyzed for the mean fluorescence intensity of GFP, but not sorted for further studies.

Gating strategy	First gating: FSC1-Height, SSC1-Height; second gating: FSC1-Height, FSC1-Width; then the fluorescence intensity was analyzed.

☒ Tick this box to confirm that a figure exemplifying the gating strategy is provided in the Supplementary Information.
