# Supplementary material for: VWCE modulates amino acid-dependent mTOR signaling and coordinates with KICSTOR to recruit GATOR1 to the lysosomes
Source: Nat Commun. 2023 Dec 20;14:8464. doi: 10.1038/s41467-023-44241-8 (PMC10733324; doi:10.1038/s41467-023-44241-8)

Figure 1

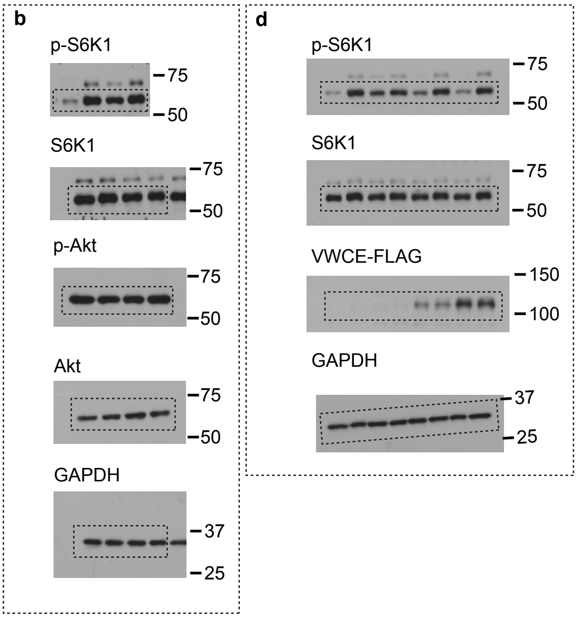
b
p-S6K1
75
50
S6K1
75
50
p-Akt
75
50
Akt
75
50
GAPDH
37
25
d
p-S6K1
75
50
S6K1
75
50
VWCE-FLAG
150
100
GAPDH
37
25

Figure 2

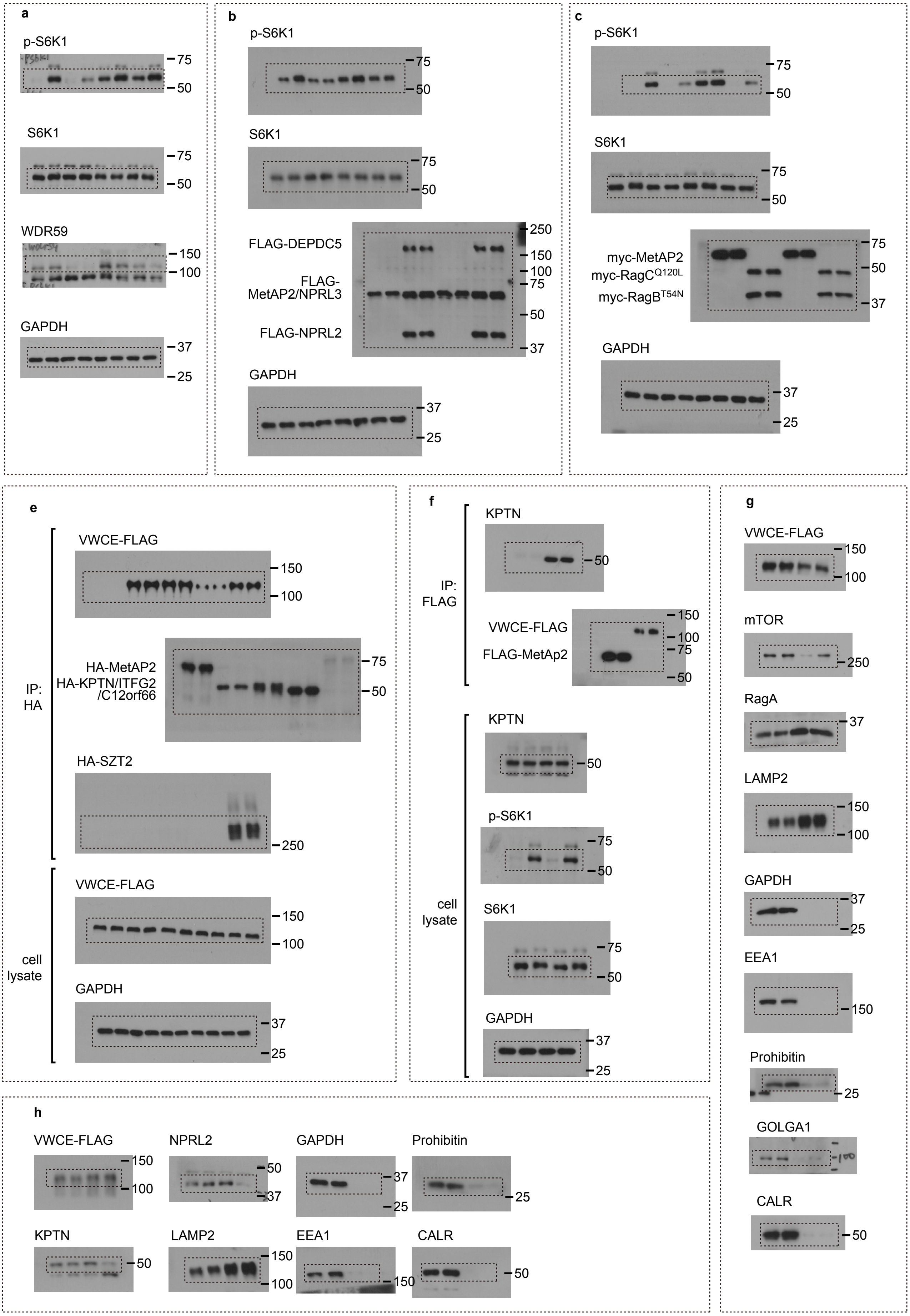
a
p-S6K1
S6K1
WDR59
GAPDH
b
p-S6K1
S6K1
FLAG-DEPDC5
FLAG-MetAP2/NPRL3
FLAG-NPRL2
GAPDH
c
p-S6K1
S6K1
myc-MetAP2
myc-RagC^Q120L
myc-RagB^T54N
GAPDH
e
IP: HA
VWCE-FLAG
HA-MetAP2
HA-KPTN/ITFG2/C12orf66
HA-SZT2
cell lysate
VWCE-FLAG
GAPDH
f
IP: FLAG
KPTN
VWCE-FLAG
FLAG-MetAp2
cell lysate
KPTN
p-S6K1
S6K1
GAPDH
g
VWCE-FLAG
mTOR
RagA
LAMP2
GAPDH
EEA1
Prohibitin
GOLGA1
CALR
h
VWCE-FLAG
NPRL2
GAPDH
Prohibitin
KPTN
LAMP2
EEA1
CALR
250
150
100
75
50
37
25

Figure 3

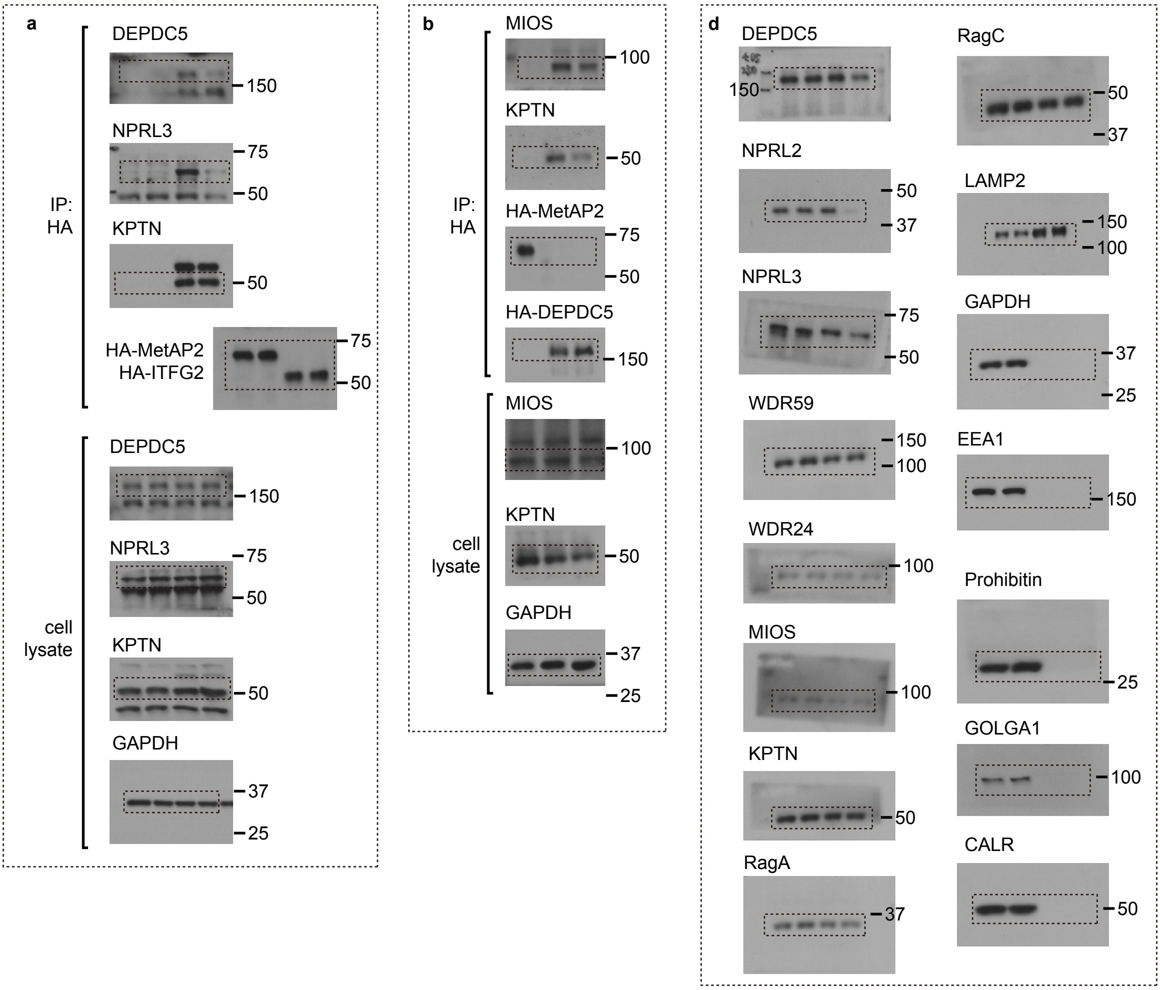
a
DEPDC5
150
NPRL3
75
50
KPTN
50
HA-MetAP2
HA-ITFG2
75
50
IP:
HA
DEPDC5
150
NPRL3
75
50
KPTN
50
GAPDH
37
25
cell
lysate
b
MIOS
100
KPTN
50
HA-MetAP2
75
50
HA-DEPDC5
150
IP:
HA
MIOS
100
KPTN
50
GAPDH
37
25
cell
lysate
d
DEPDC5
150
NPRL2
50
37
NPRL3
75
50
WDR59
150
100
WDR24
100
MIOS
100
KPTN
50
RagA
37
RagC
50
37
LAMP2
150
100
GAPDH
37
25
EEA1
150
Prohibitin
25
GOLGA1
100
CALR
50

Figure 4

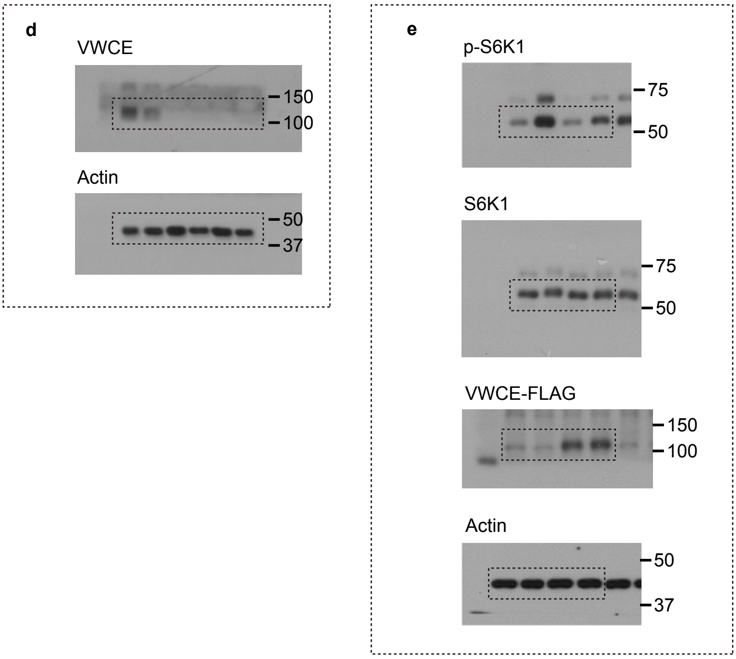
d
VWCE
150
100
Actin
50
37
e
p-S6K1
75
50
S6K1
75
50
VWCE-FLAG
150
100
Actin
50
37

Supplementary Figure 1

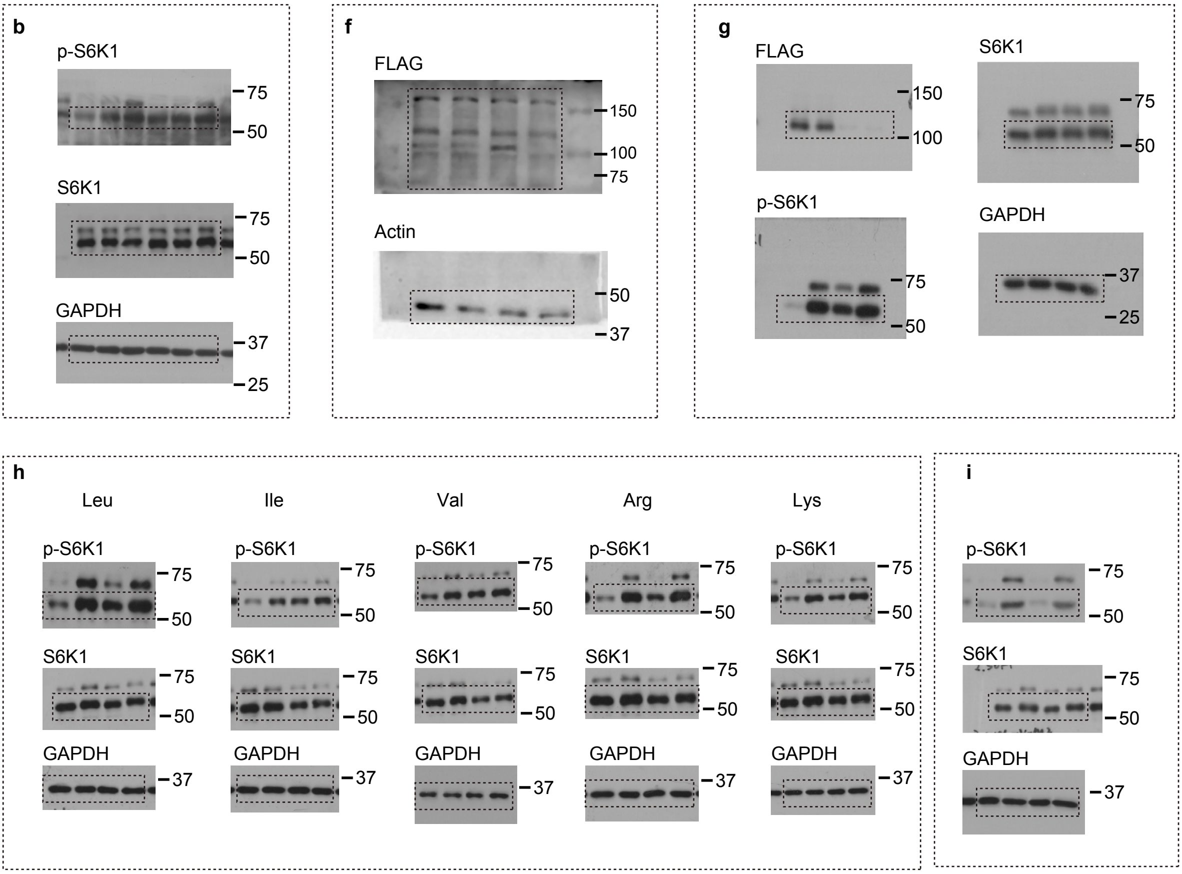

Supplementary Figure 3

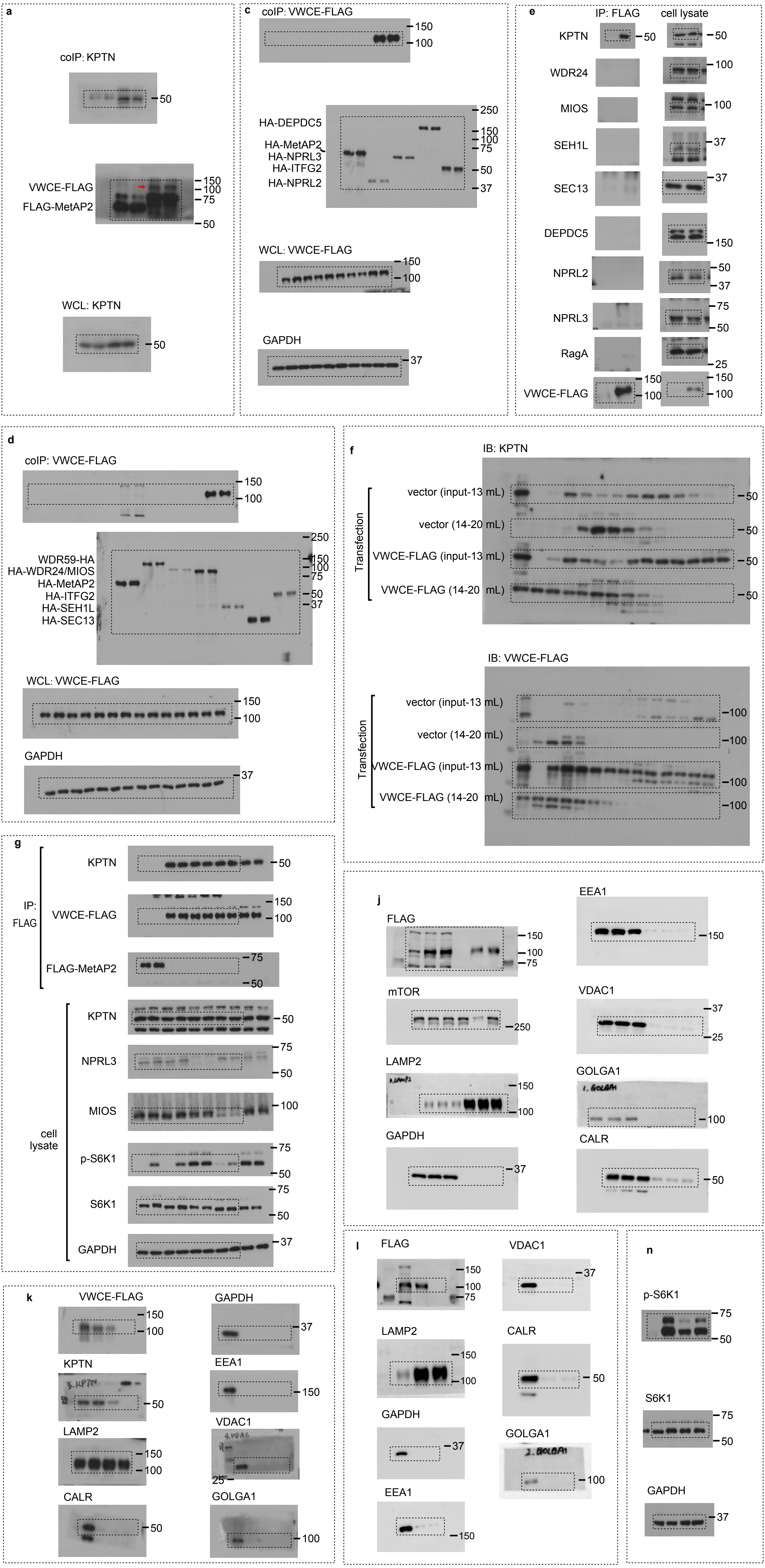

Supplementary Figure 4

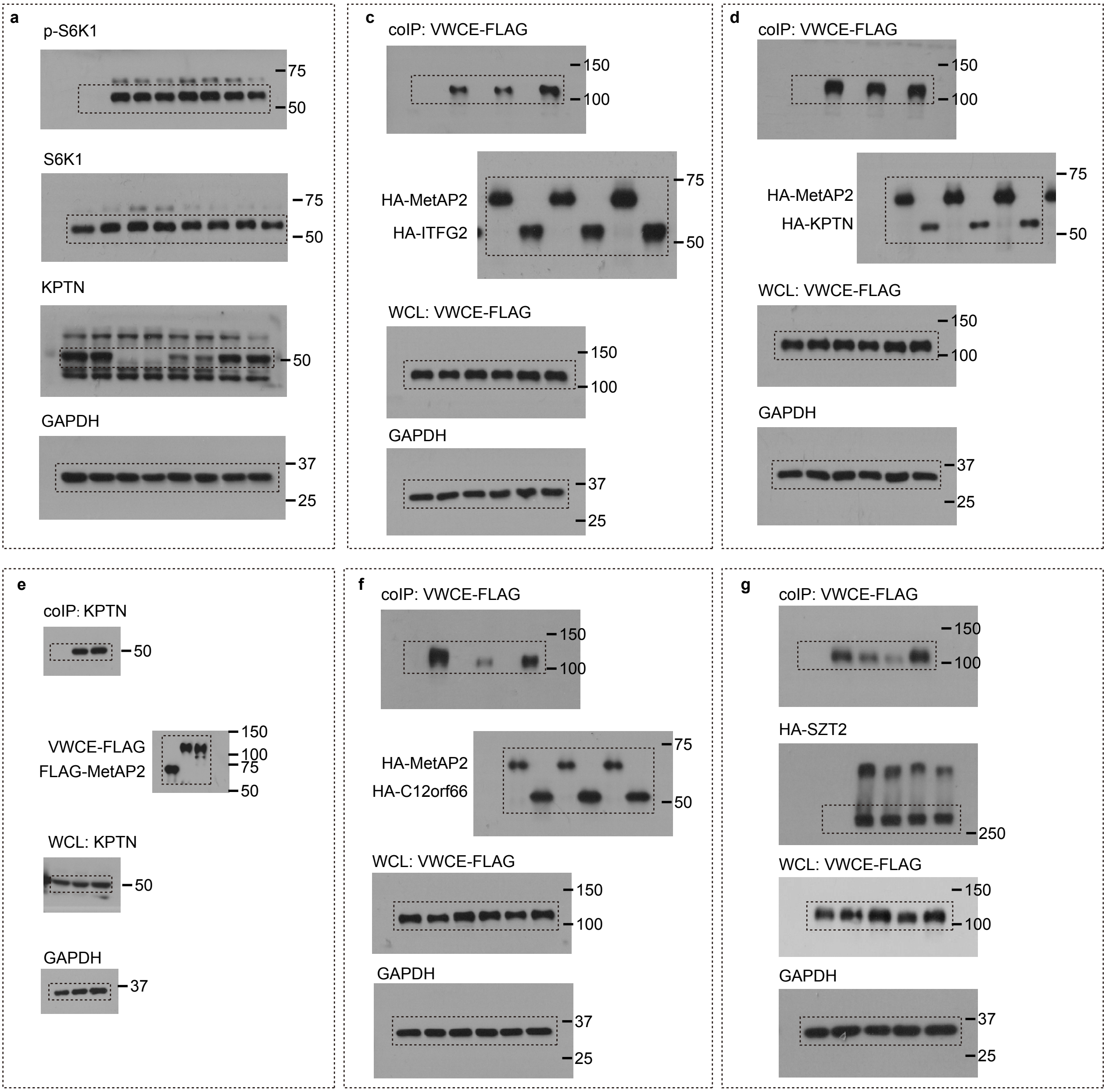

Supplementary Figure 5

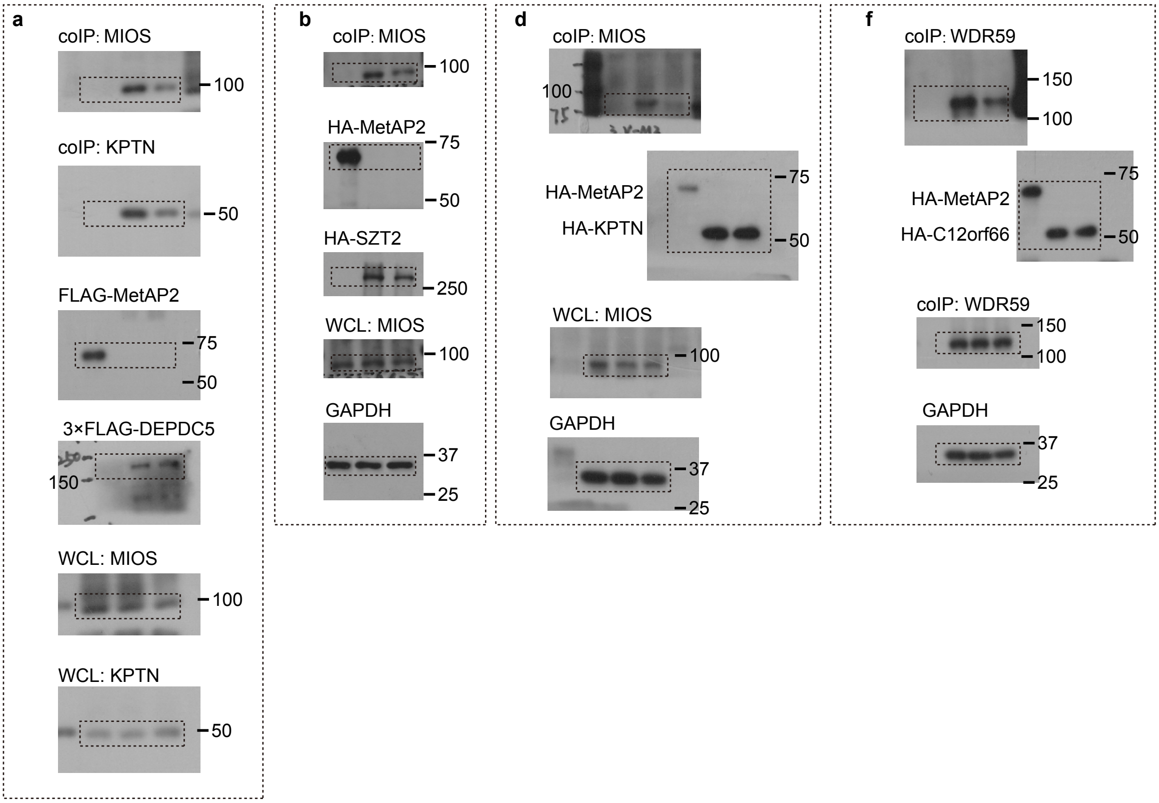

Supplementary Figure 6

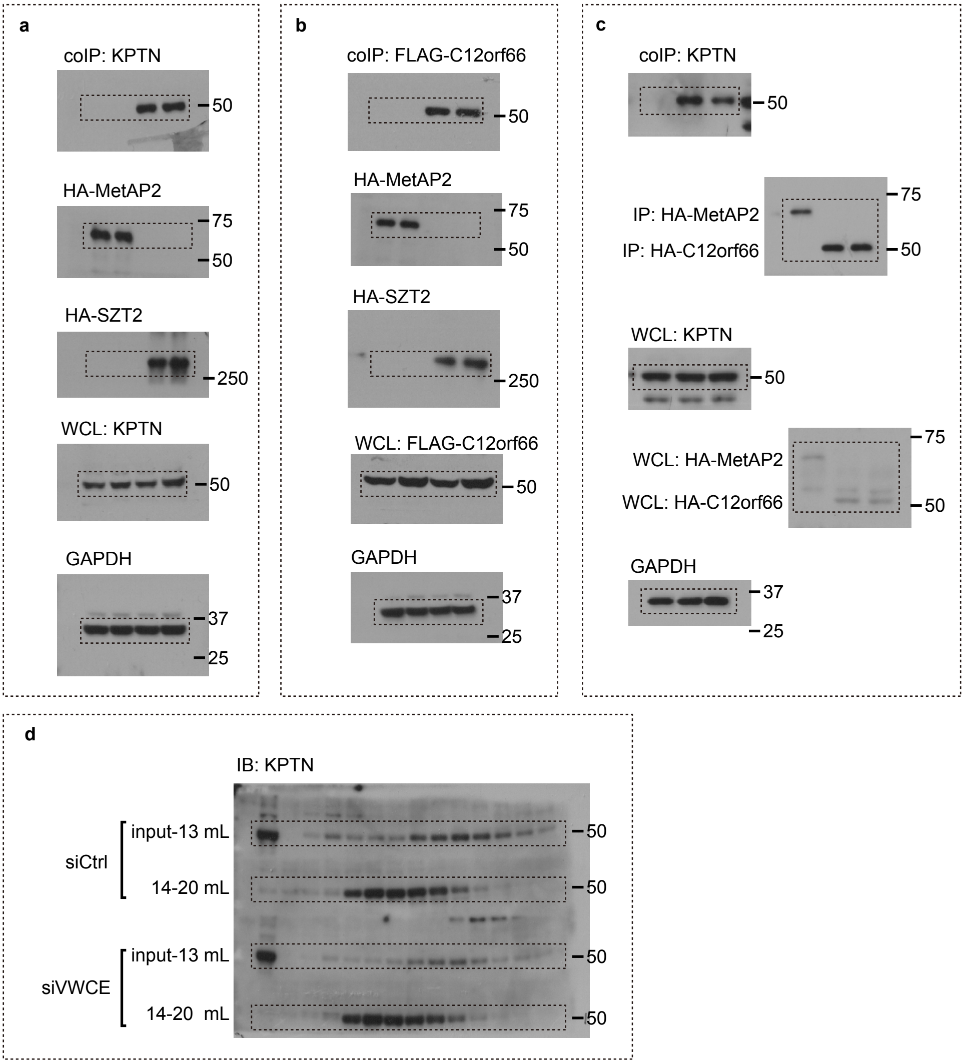

Supplementary Figure 7

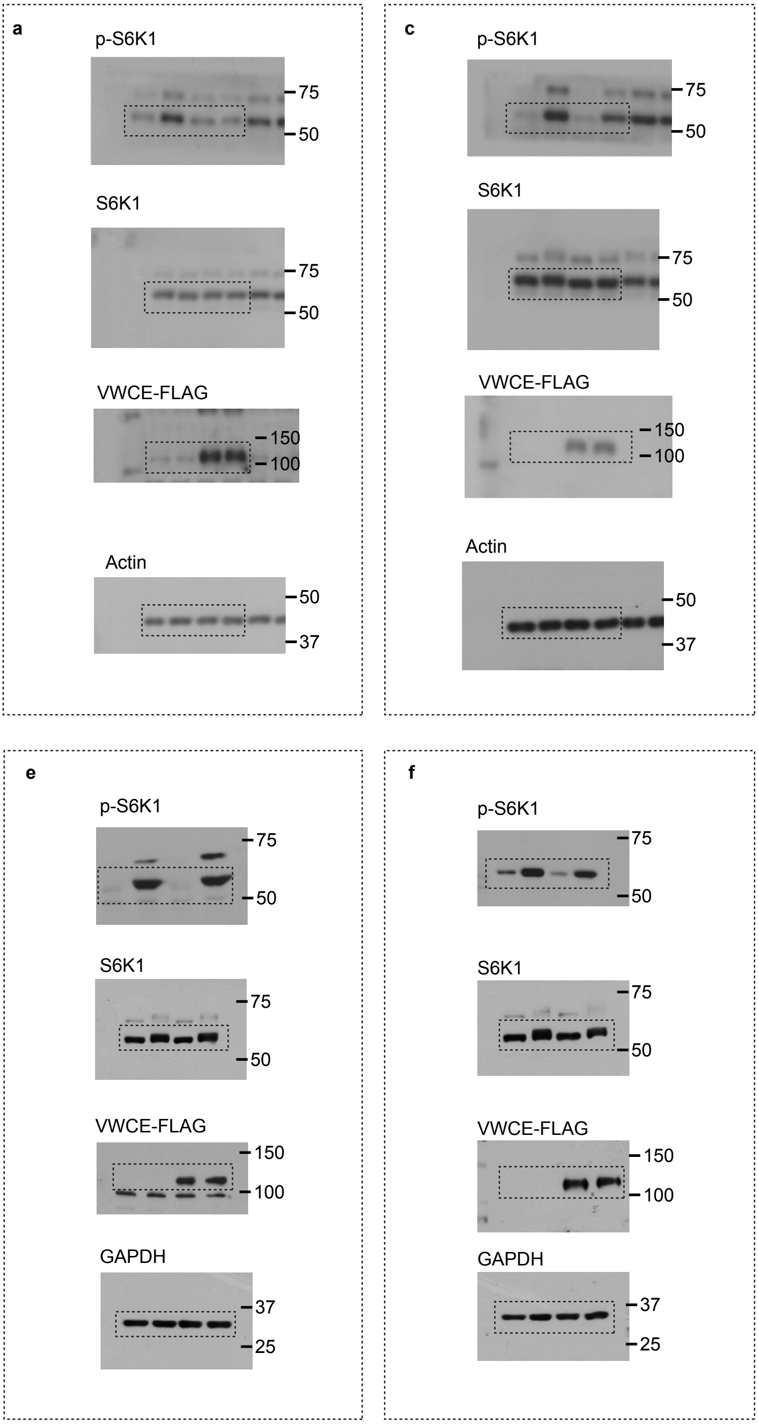

Supplement: Supplementary file 4 — Source Data [file 41467_2023_44241_MOESM4_ESM.zip › Source Data file/Source Data-Unprocessed western blots.pdf]
